# Dopamine Promotes Motor Cortex Plasticity and Motor Skill Learning via PLC Activation

**DOI:** 10.1371/journal.pone.0124986

**Published:** 2015-05-04

**Authors:** Mengia-Seraina Rioult-Pedotti, Ana Pekanovic, Clement Osei Atiemo, John Marshall, Andreas Rüdiger Luft

**Affiliations:** 1 Clinical Neurorehabilitation, Department of Neurology, University of Zurich, Zurich, Switzerland; 2 Rehabilitation Initiative and Technology Center Zurich (RITZ), Zurich, Switzerland; 3 Division of Vascular Neurology and Neurorehabilitation, Department of Neurology, University Hospital Zürich, Zurich, Switzerland; 4 Department of Molecular Pharmacology, Physiology and Biotechnology, Brown University, Providence, Rhode Island, United States of America; Queens College and the Graduate Center, CUNY, UNITED STATES

## Abstract

Dopaminergic neurons in the ventral tegmental area, the major midbrain nucleus projecting to the motor cortex, play a key role in motor skill learning and motor cortex synaptic plasticity. Dopamine D1 and D2 receptor antagonists exert parallel effects in the motor system: they impair motor skill learning and reduce long-term potentiation. Traditionally, D1 and D2 receptor modulate adenylyl cyclase activity and cyclic adenosine monophosphate accumulation in opposite directions via different G-proteins and bidirectionally modulate protein kinase A (PKA), leading to distinct physiological and behavioral effects. Here we show that D1 and D2 receptor activity influences motor skill acquisition and long term synaptic potentiation via phospholipase C (PLC) activation in rat primary motor cortex. Learning a new forelimb reaching task is severely impaired in the presence of PLC, but not PKA-inhibitor. Similarly, long term potentiation in motor cortex, a mechanism involved in motor skill learning, is reduced when PLC is inhibited but remains unaffected by the PKA inhibitor. Skill learning deficits and reduced synaptic plasticity caused by dopamine antagonists are prevented by co-administration of a PLC agonist. These results provide evidence for a role of intracellular PLC signaling in motor skill learning and associated cortical synaptic plasticity, challenging the traditional view of bidirectional modulation of PKA by D1 and D2 receptors. These findings reveal a novel and important action of dopamine in motor cortex that might be a future target for selective therapeutic interventions to support learning and recovery of movement resulting from injury and disease.

## Introduction

Dopaminergic neurotransmission is involved in a large variety of physiological functions including voluntary motor activity, reward control, learning and cognition[1,2,3]. Dysfunction of the dopaminergic system has been linked to pathologies such as schizophrenia, drug addiction and Parkinson’s disease[3]. Dopamine (DA) modulates glutamatergic and GABAergic neurotransmission via D1-like and D2-like receptor subclasses to exert opposing physiological effects. In the prefrontal cortex (PFC) DA modulates pyramidal cell excitability directly and indirectly through its actions on local GABAergic interneurons[4]. D1 stimulation increases interneuron excitability leading to increased evoked and spontaneous inhibitory postsynaptic currents (IPSCs) in pyramidal neurons, while D2 stimulation reduces IPSCs[5,6]. Typically, D1-like receptors (D1, D5) activate adenylyl cyclase, whereas DA D2-like receptors (D2, D3, D4) inhibit adenylyl cyclase[7]. In the striatum, D1 receptors are positively coupled to adenylyl cyclase-PKA resulting in enhanced excitability in striatonigral medium spiny neurons (MSNs), whereas D2 receptor signaling exerts the opposite effect in striatopallidal MSNs[7]. DA receptor activation also plays a critical role in modulating synaptic strength of glutamatergic inputs[8,9]. D1 receptors are required for the induction of LTP at glutamatergic synapses in direct pathway MSNs[8,9], and at hippocampal synapses[10,11]. Activation of D2 receptors on striatal MSNs of the indirect pathway is necessary for long-term depression (LTD)[8,9]. DA receptors also have the ability to form heterooligomers which form the starting point of a different signaling pathway[12]. The D1-D2 heteromer has been reported to be coupled to G_q/11_ to activate PLC which triggers intracellular Ca^2+^ release, and phosphorylation of calcalcium/calmodulin-dependent protein kinase II (CaMKII)[13], which is known to play a key role in both long-term potentiation (LTP) and LTD of synaptic transmission[14].

In Parkinson’s disease (PD), degeneration of the DA neurons projecting to the neocortex leads to a 70% reduction of DA fibers within the primary motor cortex (M1) and other frontal cortical areas[1]. Successful motor skill learning and synaptic plasticity in M1 requires intact dopaminergic signaling within M1[15]. Dopaminergic projections to M1 originate predominantly in the midbrain‘s ventral tegmental area (VTA)[16]. Destruction of dopaminergic neurons in the VTA by 6-hydroxydopamine (6-OHDA) depletes dopaminergic terminals in M1 and impairs motor skill acquisition[15,16]. Additionally, LTP in M1, a mechanism involved in skill acquisition[17,18] is reduced by both D1 and D2 receptor antagonists[15]. The parallel effects of antagonists cannot be explained by the traditional mechanism of opposing D1 and D2 receptor monomer effects on the cAMP-PKA pathway. Here we tested the hypothesis that DA influences M1 synaptic plasticity and motor skill acquisition via activation of the intracellular PLC signaling pathway. We show that inhibition of PLC but not of PKA prevents the acquisition of a reaching skill and impairs LTP in M1. PLC agonist treatment abrogates the learning deficit and LTP impairment induced by DA antagonists in M1.

## Material and Methods

### Animals

All experiments were performed with adult male Long-Evans rats (8–10 weeks, 250–350 g) housed in pairs on a 12/12-hr light/dark cycle. Experiments and procedures were conducted according to the German and Swiss national guidelines and approved by the Animal Care Committee of the State of Baden Württemberg (Germany) or the Committee for Animal Experimentation of the Kanton of Zürich (Switzerland). Most chemicals were purchased from Tocris bioscience (Bristol, UK): H-89 hydrochloride (PKA inhibitor), U-73122 (active) and U-73343 (inactive) (PLC inhibitor), m-3m3fbs (PLC agonist), SCH23390 hydrochloride (D1 antagonist), bicuculline methiodide (GABAA antagonist, used for LTP induction). Raclopride tartrate salt (D2 antagonist) was purchased from Sigma-Aldrich Chemie GmbH (Steinheim, Germany).

### Motor skill training

Training sessions were performed at the beginning of the dark phase. Animals were food-restricted for 24 hr prior to the first pre-training session. During training animals were kept slightly over their initial weight (336.7 ± 31.2 g) by providing 50 mg/kg of standard lab diet after each training session. Water was given ad libitum. For all experiments, litter-mates were equally assigned to experimental groups.

The single pellet reaching task was performed as previously described [20]. The training cage had a vertical window with a sliding door in the front wall and a light sensor in the rear wall. Nose-poking the sensor opened the sliding door giving access to single food pellets (45 mg, Bio-serve, Frenchtown, NJ, USA). During the 5 day shaping period rats retrieved pellets with their tongue from a small horizontal board 0.5 cm distance from the window. After shaping, single food pellets were placed on a post with a slightly larger diameter than the pellets in 1.5 cm distance from the window. Thus, pellets were accessible only with the forelimb. The first training session consisted of 50 door openings (= trials) and was used to determine forelimb preference which was necessary for implantation of cannulas and needles in the contralateral hemisphere. For all subsequent training sessions which consisted of 100 trials the post was shifted to one side of the window to facilitate reaching. To retrieve the pellet rats had to extend the forelimb towards the pellet, grasp, retrieve and bring the pellet to the mouth[19]. Each reaching trial was scored as “successful” or “unsuccessful”. The success rate was defined as the ratio of the number of successful trials and the total number of trials per session, i.e. 100. The latency between pellet removal and subsequent door opening was used as an index of motivation [20].

### Surgical Procedures

All surgical procedures were performed under ketamine (70 mg/kg, i.p.) and xylazine anesthesia (5 mg/kg, i.p.) with the rats fixed in a stereotactic frame (Stoelting Co., Wood Dale, IL, USA). Additional ketamine doses were administered as needed. Body temperature was monitored using a heating pad. Buprenorphin (0.01 mg/kg, i.p.) was given after surgery for pain relief. All permanent implants were anchored onto the skull with two screws (2 mm diameter) placed in the frontal and occipital skull. Screws and guide cannulas were connected and stabilized using bone cement (FlowLine, Heraus Kulzer, Dormagen, Germany).

#### Cannula implantation

For acute drug injections into M1, PLC- and PKA inhibitors, DA antagonists and PLC agonist, were administered via guide cannula systems (15 mm long, Unimed SA, Lausanne, Switzerland) implanted into the center of the M1 forelimb representation (3 mm lateral, 1 mm anterior to bregma, 900 μm below dura). Prior to implantation forelimb preference for reaching was determined (see above) and cannulas were implanted into the contralateral hemisphere. After 3 days of recovery, rats were trained for 7 successive days. Drugs were injected 30 min before the training session on days 2 and 3 (34 Gauge needle, Hamilton Bonaduz AG, Switzerland). A volume of 0.5 μl was injected over 90 sec using a microsyringe (5 μl, Hamilton Bonaduz AG, Switzerland) connected via plastic tubing (10 cm, PE40 Plastics One, Roanoke, VA USA) to a microinjection pump (Nano-injector, Stoelting Co., Wood Dale, IL, USA). Following the last training session cannulas were removed and location was verified histologically.

#### Osmotic minipump implantation

For continuous drug application into M1, PLC- and PKA inhibitors, and vehicle were administered via osmotic minipumps (0.25 μl/hr, 100 μl volume, model 1002, Alzet, Cupertino, CA, USA). Prior to minipump implantation the forelimb preference was determined and the injection needle was implanted into the M1 forelimb area of the contralateral hemisphere. Minipump reservoirs were implanted subcutaneously in the neck area and connected to the implanted needle via plastic tubing. The pump was loaded with either PLC or PKA inhibitors followed by a vehicle solution. Double-loading ensured that vehicle was released during the recovery period from surgery and inhibitors only during the training period. After the last training session minipumps were explanted and their location verified histologically.

#### Histological verification

The positioning of cannulas and needles for minipumps was verified histologically using Nissl staining. No animal had to be excluded because of cannula or needle misplacement.

### Drug applications

For cannula injections, PKA inhibitor (H89), PLC agonist (m-3m3fbs) and DA antagonists (SCH23390 and raclopride) were dissolved in 0.9% saline, active and inactive PLC inhibitor (U73122 and U73343) in DMSO at 100 μM, and injected in volumes of 0.5 μl. For minipump injections, PLC- and PKA inhibitor were continuously administered at 100 μM concentration for the entire training period (0.25 μl/hr, 100 μl volume). For electrophysiological recordings, the PLC agonist (25 μM) and the PLC inhibitor (10 μM) were dissolved in DMSO (0.005%). The PKA inhibitor (20 μM), the D1 and the D2 antagonists (5 μM) were dissolved in saline. Drugs were added to the solution superfusing the slice.

### *In vitro* slice preparation

Deeply anesthetized rats (pentobarbital, 50 mg/kg) were decapitated, their brains quickly removed and immersed in cold (5–7°C), oxygenated (95% O2/5% CO2) artificial cerebrospinal fluid (ACSF) containing (in mM): 126 NaCl, 3 KCl, 1.25 NaH2PO4, 1 MgSO4, 2 CaCl2, 26 NaHCO3, 10 dextrose. Coronal slices (500 μm) including the M1 forelimb area (1.5–3.5 mm anterior to Bregma, 2–4 mm lateral) of both hemispheres were prepared using a vibratome. Slices were transferred to a temperature controlled (34 ± 0.5°C) interface chamber and superfused with oxygenated ACSF at a rate of 1–2 ml/min. Slices were allowed to recover for at least one hour prior to the start of recordings.

### Stimulation and recording

To record intracortical horizontal connections in brain slices, concentric bipolar stimulation electrodes (FHC, Main St Bowdoin, USA) were positioned mirror symmetrically in layer II/III of each hemisphere 2–2.5 mm lateral to the midline. Recording electrodes were placed 500 μm lateral to the stimulation electrodes. Extracellular field potentials (FP) were evoked by 0.2 ms pulses at 0.03 Hz and recorded simultaneously in both hemispheres. Stimulation intensity was adjusted until a response of 0.2 mV was recorded, which was defined as the threshold intensity. Synaptic strength was determined by generating input-output relationships at multiples of threshold intensity.

### LTP induction

The stimulus intensity eliciting 50% of the maximum peak amplitude was used for all measurements before and after LTP induction. Baseline amplitudes were recorded using single stimuli applied every 30 sec. Following a 30-min stable baseline period, LTP was induced by theta burst stimulation (TBS), consisting of 10 trains at 5 Hz, each train composed of 4 (200 μsec) pulses at 100 Hz, repeated 5 times every 10 sec. During TBS the stimulation intensity was doubled. TBS was applied immediately after transient, local application of the GABAA receptor antagonist bicuculline methiodide (3.5 mM) at the field potential recording site until response amplitude increased to 150–200% of baseline.

### Statistical Analysis

Repeated measures analysis of variance was used to explore the effects of drug injections on reaching success rate or intertrial latencies (using Graphpad Prism version 6, Graphpad Inc, USA). The within-subjects variable *training day*, the between-subjects variable *group* and their interaction were included as independent predictors. If group or the interaction *group* x *training day* were significant, post hoc tests were performed using Dunett’s test for comparison with the control group. Two-tailed probability less or equal to 5% was considered significant.

## Results

### PLC inhibitor impairs motor skill acquisition and M1 synaptic plasticity

**Fig 1:**
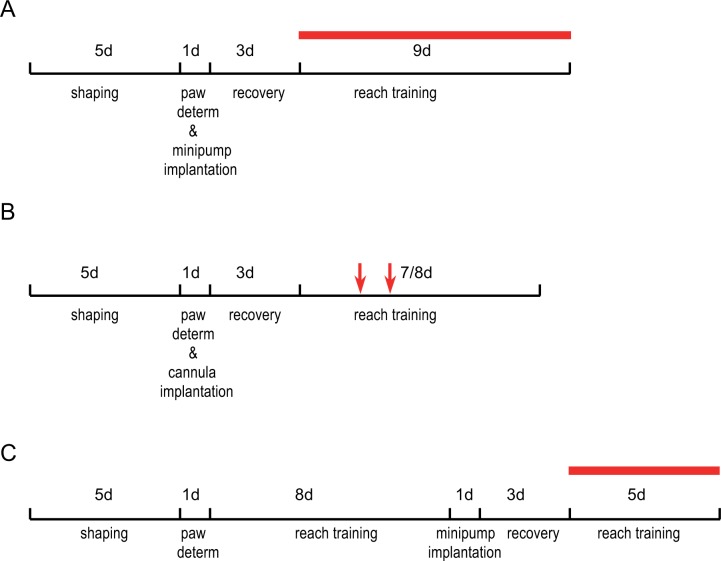
Experimental design. (a) Timeline for experiments shown in Fig 2A using continuous drug release via minipump (red bar) during motor skill training. (b) Timeline for experiments shown in Fig 2B, Fig 3A and 3B using acute drug injections on day 2 and 3 of motor skill training (red arrows). (c) Timeline for experiments shown in Fig 2 using continuous drug release via minipump (red bar) after successful acquisition of the motor task.

**Fig 2:**
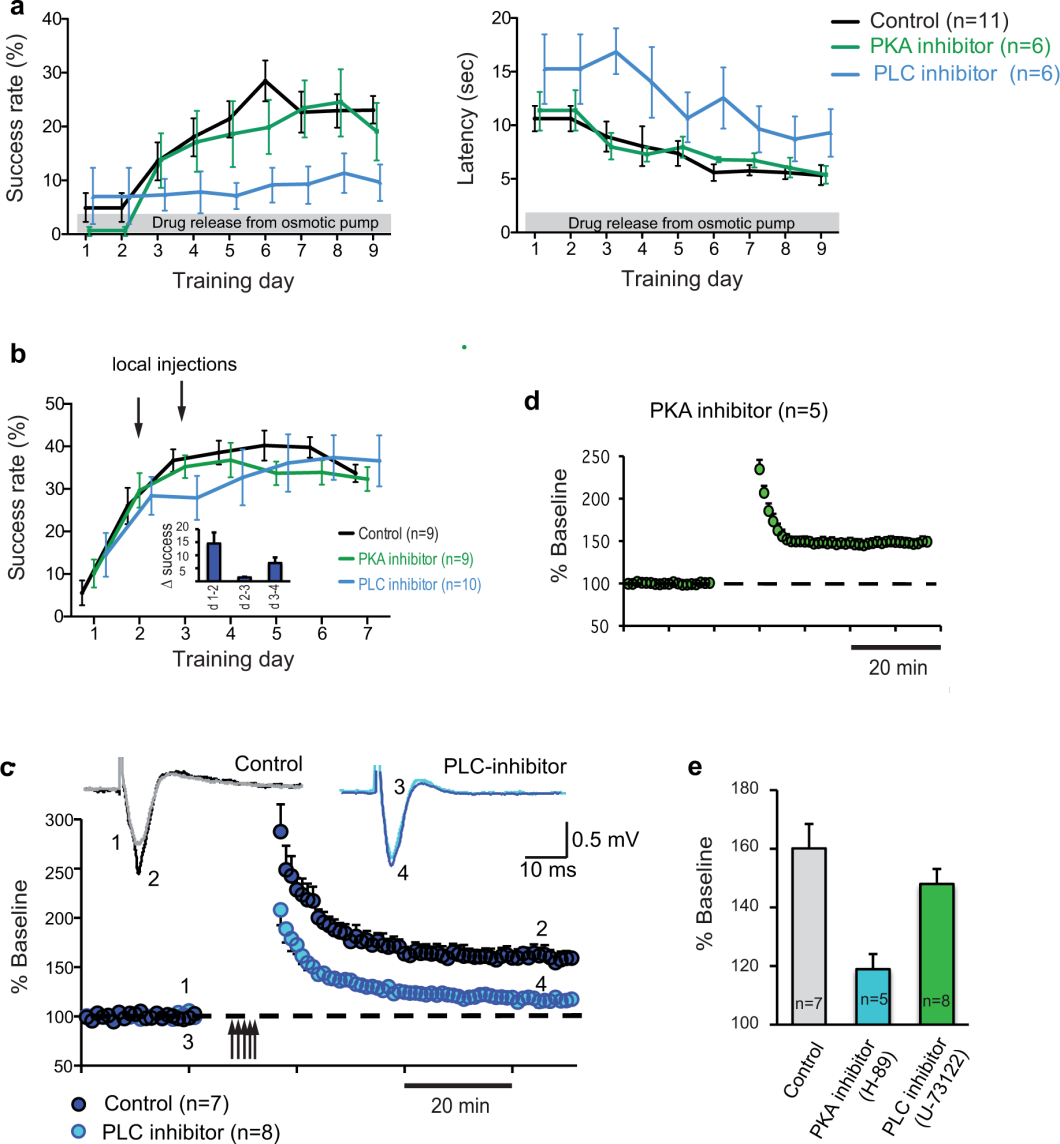
PLC inhibitor impairs motor skill acquisition and the potential for M1 synaptic plasticity. (a) Motor skill acquisition was assessed in rats that received continuous intracortical injection (grey bar) of PLC inhibitor U-73122 (blue), PKA inhibitor H-89 (green) and inactive PLC inhibitor U-73343 (black) into the M1 forelimb area via osmotic minipumps. The success rate and the latency, a measure of motivation, were impaired in the presence of PLC but not PKA inhibitor. Error bars indicate SEM. (b) Temporary application of PLC and PKA inhibitors directly to M1 on training day 2 & 3 (time of steepest learning) reveal the same effect as with continuous application. Arrows indicate local injection days. The inset illustrates the rate of learning from day 1–2, day 3–4, and day 4–5 indicating a lack of improvement from day 2–3. (c) Maximum synaptic strength (LTP saturation) by repeated induction of LTP (multiple arrows) in layer II/III horizontal connections in brain slices. Peak amplitudes were assessed in the M1 forelimb area in ACSF (dark blue) and in the presence of PLC inhibitor U-73122 (light blue). Each FP trace (insets) represents an average of 10 individual responses at times indicated by numbers. (d) Maximum LTP in the presence of PKA inhibitor is not significantly different from control LTP. Arrows indicate multipe LTP attempts. (e) Summary histogram of LTP. Grey: Control; green: PKA inhibition; blue: PLC inhibition.

To establish whether DA activates intracellular PKA and/or PLC signaling pathways in M1 specific inhibitors were continuously infused directly into the M1 forelimb area of adult rats via osmotic minipumps while the animals were learning a new motor skill (Fig 1A). Rats were trained to reach with a single forepaw through a small aperture to reach and grasp single food pellets for 8 successive days. Learning curves as illustrated in Fig 2 indicate a significant impairment of skill acquisition in animals that received intracortical infusion of the PLC inhibitor U73122 while infusion of PKA inhibitor H89 had no effect (N = 6 per inhibitor, N = 12 controls; interaction of group by time: F (16, 168) = 2.557, p = 0.0015, significant post-hoc Dunnetts‘s tests for comparison between PLC inhibitor and control for training session 5: mean difference -14.26 (CI -26.55 to -1.977); session 6: -19.30 (CI -31.59 to -7.016); session 7: -13.35 (CI -25.64 to -1.067); and session 9:, -13.43 (CI -25.71 to -1.141); Fig 2A left). Time intervals between reaching trials were used as a measure of motivation. They were significantly longer in all training sessions in PLC inhibitor-treated rats as compared with vehicle-treated controls but were unaffected in PKA inhibitor-treated rats (main effect of group: F (2, 20) = 7.398, p = 0.0039, significant post-hoc Dunnett‘s tests for comparison between PLC inhibitor and control: session 3: mean difference 7.910 (CI 2.891 to 12.93); session 4: 5.970 (CI 0.9507 to 10.99); and session 6: 6.978 (CI 1.959 to 12.00); Fig 2A right). Similar results were achieved when the drugs were applied via implanted cannulas during the steepest part of the learning curve, on day 2 and 3 of skill training (30 minutes prior to training; Fig 1B). Skill acquisition was impaired in the presence of PLC- but not PKA inhibitor compared to control rats (Fig 2B). This data set revealed a significant interaction of group and time (F (12, 150) = 1.980, p = 0.0297), but post hoc tests comparing the groups individually to control were not significant (p>0.8). There were no differences between groups with respect to the time intervals between reaching trials. The inset in Fig 2B emphasizes the lack of success improvement between days 2 and 3.

Previous studies have shown that motor skill acquisition is accompanied with increases in synaptic strength[17], and LTP occlusion[18], in M1 horizontal connections. Because motor skill learning requires intracellular PLC signaling we tested the hypothesis that PLC activation is also necessary for learning-induced synaptic strengthening. This was accomplished by recording maximum synaptic strength (LTP saturation) in layer II/III horizontal connections in the M1 forelimb area of brain slices in control and in the presence of PLC and PKA inhibitors. LTP was induced by multiple attempts of theta burst stimulation preceded by local transient application of bicuculline. Results show that LTP saturation was significantly reduced when PLC was blocked with U-73122 (117.2±4.8%, N = 8), as compared to the control condition (160.1±8.34%, N = 7, p = 0.0012, Fig 2C). Superfusion of slices with the PKA inhibitor H-89 resulted in LTP that was not significantly different from control LTP (148.5%±5.1%, N = 5, p = 0.31, Fig 2D), but significantly different from LTP in the presence of the PLC inhibitor (p = 0.0027). These results suggest that PLC but not PKA signaling is required for motor skill acquisition and synaptic plasticity. LTP results are summarized in Fig 2E.

### PLC inhibitor has no effect on movement execution and M1 synaptic transmission

**Fig 3:**
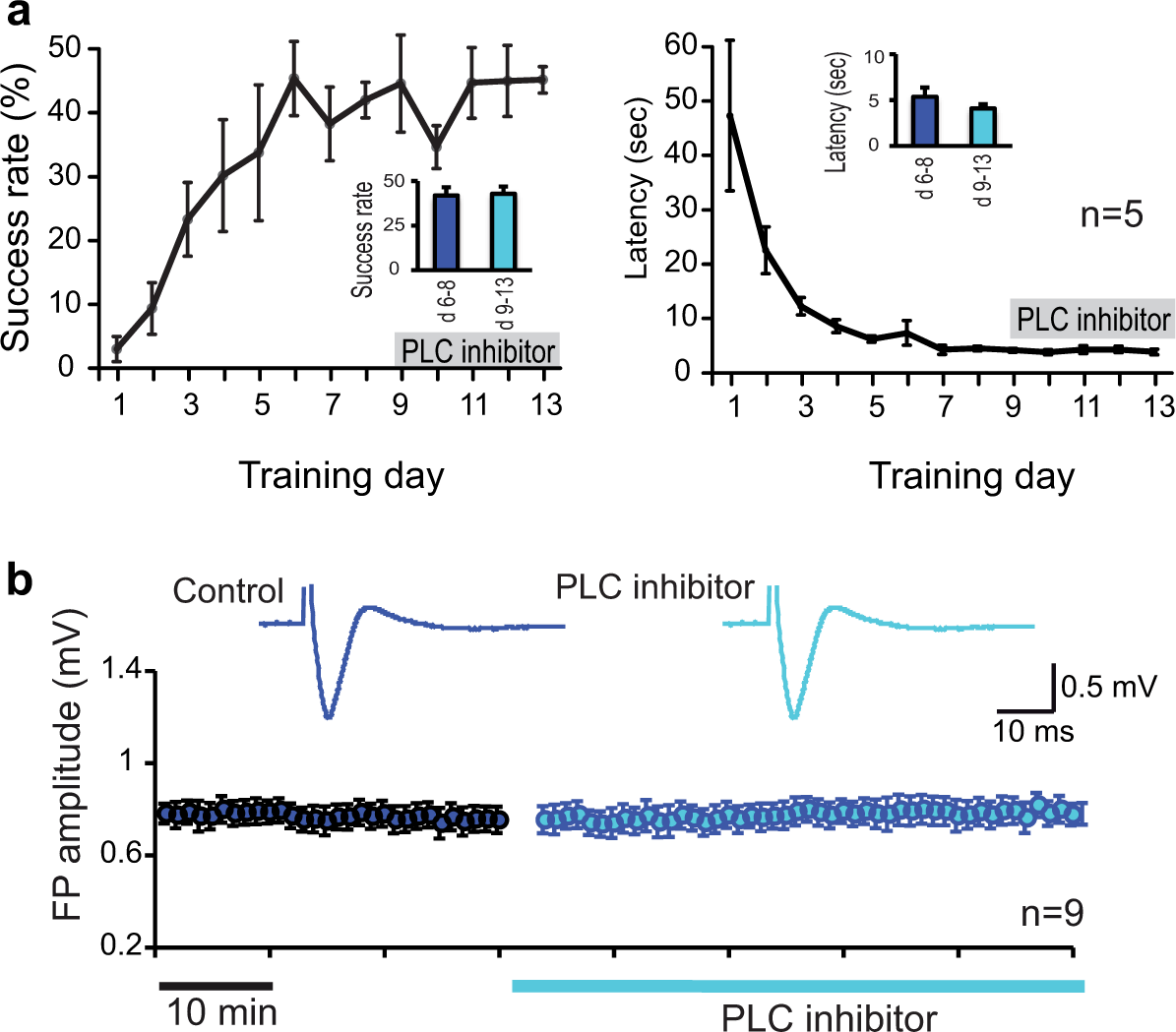
PLC activation is not necessary for the execution of an already acquired task and does not affect baseline synaptic transmission. (a) Continuous intracortical infusion of PLC inhibitor (grey bar) for 5 days following successful acquisition of the skilled reaching task did not affect success rate or intertrial latency. Insets illustrate mean±SEM before (day 6–8) and following (day 9–13) treatment with PLC inhibitor indication a lack of change. Error bars indicate SEM. (**b**) Amplitudes and shapes of extracellular field potentials (FP) in layer II/III horizontal connections of the M1 forelimb area remained unchanged in the presence of PLC inhibitor. Traces are examples of extracellular FPs at baseline stimulation intensity before (control, dark blue) and after inhibitor application (light blue).

To determine whether PLC signaling is also involved in movement execution, rats received continuous infusion of PLC inhibitor U-73122 into the M1 forelimb area after having acquired the task successfully and learning curves had reached the asymptotic phase (Fig 1C). Reaching performance was not affected when PLC was blocked (N = 5, t-test comparing the means of sessions 6–8 and sessions 9–13, t(4) = 0.2820, p = 0.79, Fig 3A left). Likewise, the intertrial latencies remained unaffected (t(4) = 1.907, p = 0.13, Fig 3A right), suggesting that PLC signaling is only necessary for skill learning but not for movement execution after a skill had been acquired successfully.

We examined whether blocking PLC and PKA affects baseline synaptic transmission by measuring evoked responses of layer II/III horizontal connections in the M1 forelimb area. Extracellular field potentials (FP) recorded before and after superfusion with the PLC inhibitor (Fig 3B) or the PKA inhibitor remained unchanged in shape and amplitude (for PLC experiments: baseline 0.788±0.050 mV, N = 9; PLC inhibitor 0,784±0.052 mV, N = 9, p = 0.48; for PKA experiments: baseline 1.15±0.048 mV, PKA inhibitor 1.16±0.049 mV, N = 5, p = 0.61). Thus, blocking intracellular PLC signaling pathway affects only synaptic plasticity but not baseline synaptic transmission.

### PLC activation prevents DA antagonist-induced learning deficit and LTP

**Fig 4:**
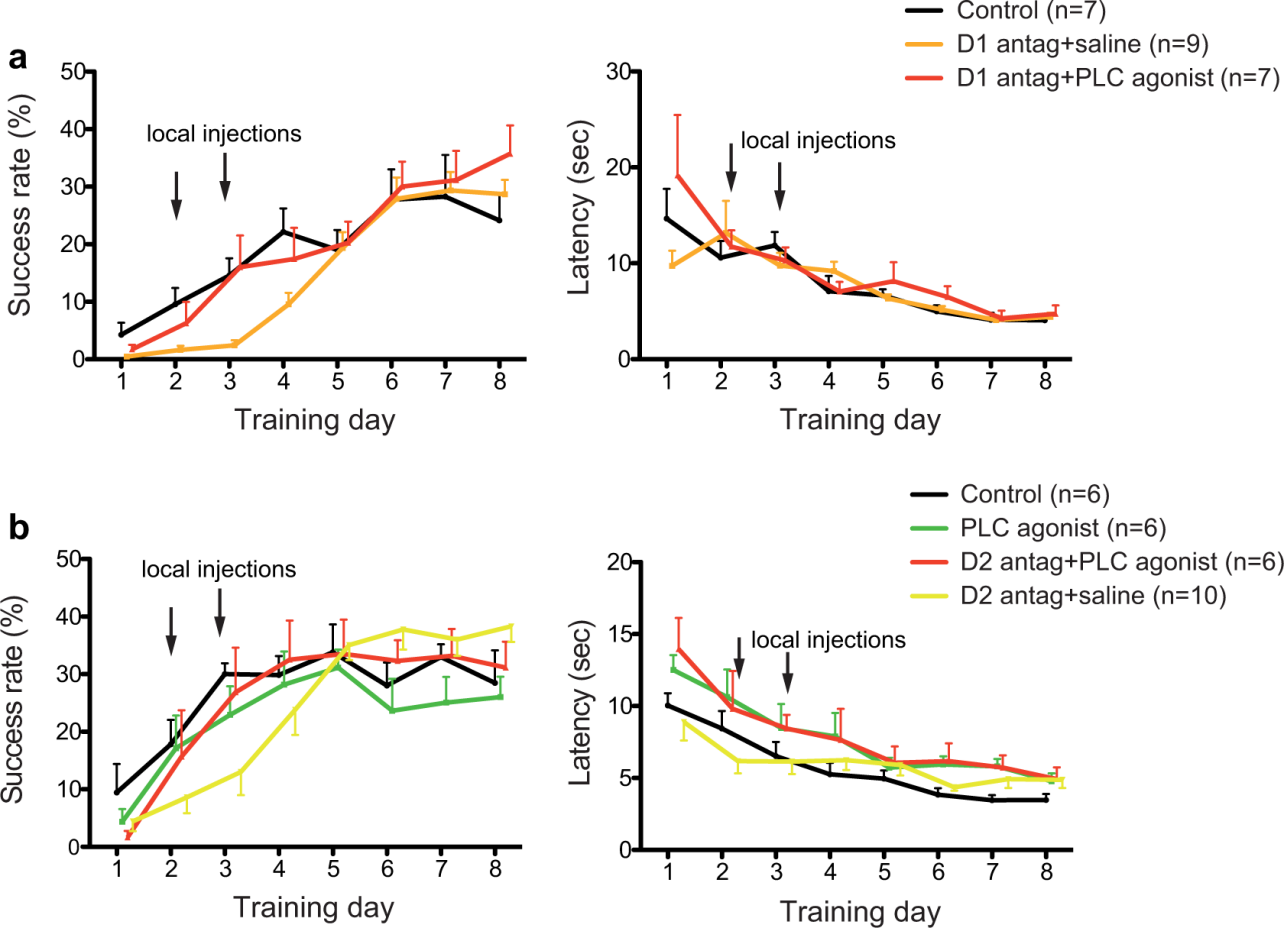
PLC activation prevents DA antagonist-induced learning deficits. (a) Co-administration of the PLC agonist m-3M3FBS and D1 receptor antagonist SCH 23390 (red) prevents SCH 23390 (orange) induced deficit in the success rate (left) compared to vehicle injected control (black). Intertrial latencies remained unaffected regardless of drug or drug combination injected (right). (b) D2 receptor antagonist raclopride (yellow) impaired skill acquisition. This impairment was prevented by PLC agonist co-administration (red). PLC agonist alone (green) had no effect on learning as compared to controls (black). Intertrial latencies remain unaffected by all treatments (right). Error bars indicate SEM. Arrows indicate days of local injections.

Previous findings showed that D1 and D2 receptor antagonists significantly impair motor skill acquisition and synaptic plasticity[15]. Similarly blocking the PLC pathway drastically reduces the capacity for skill learning and synaptic plasticity (Fig 2). As DA binding to D1 and D2 receptors can activate the PLC signaling pathway[21], we examined whether PLC activation in the presence of D1 or D2 receptor antagonists could prevent the observed learning impairment and LTP deficits. The PLC agonist m-3m3fbs was co-administered with either D1 receptor antagonist SCH23390 or D2 receptor antagonist raclopride on day 2 and 3 during motor skill training (Fig 1B). DA receptor antagonists alone significantly impaired skill learning compared to control while learning deficits were prevented by co-administration of the PLC agonist m-3m3fbs (D1 antagonist SCH23390 N = 9, SCH23390+m-3m3fbs N = 7, control N = 7, group by time interaction: F (14, 140) = 1.974, p = 0.0238, significant post-hoc Dunnett’s test between SCH 23390 and control for training session 3: mean difference -12.13 (CI -23.41 to -0.8420); and 4: -12.57 (CI -23.86 to -1.290); Fig 4A left; D2 antagonist raclopride N = 10, raclopride+m-3m3fbs N = 6, control N = 6, m-3m3fbs alone N = 6, group by time interaction: F (21, 168) = 2.645, p = 0.0003, significant post-hoc Dunnett’s test between raclopride and control for training session 3:, mean difference -17.00 (CI -30.71 to -3.286), Fig 4B left). All Dunnett’s test for comparisons between DA-antagonist+PLC-agonist groups and control were not significant. To exclude possible additive but mechanistically unrelated effects of DA antagonists and the PLC agonist, one group of rats was treated with PLC agonist alone. The learning curves of PLC agonist treated rats and vehicle treated controls were not significantly different (post-hoc Dunnett’s tests were not significant). Intertrial latencies in rats treated with DA-receptor antagonists, PLC agonist, and PLC agonist combined with DA antagonists were not significantly different from vehicle-treated controls (SCH23390 experiment group by time effect: F (14, 140) = 1.157, p = 0.34, raclopride experiment: F (21, 161) = 1.090, p = 0.36, Fig 4A and 4B right). These results are consistent with the hypothesis that DA binding to D1 and D2 stimulates the PLC signaling pathway.

**Fig 5:**
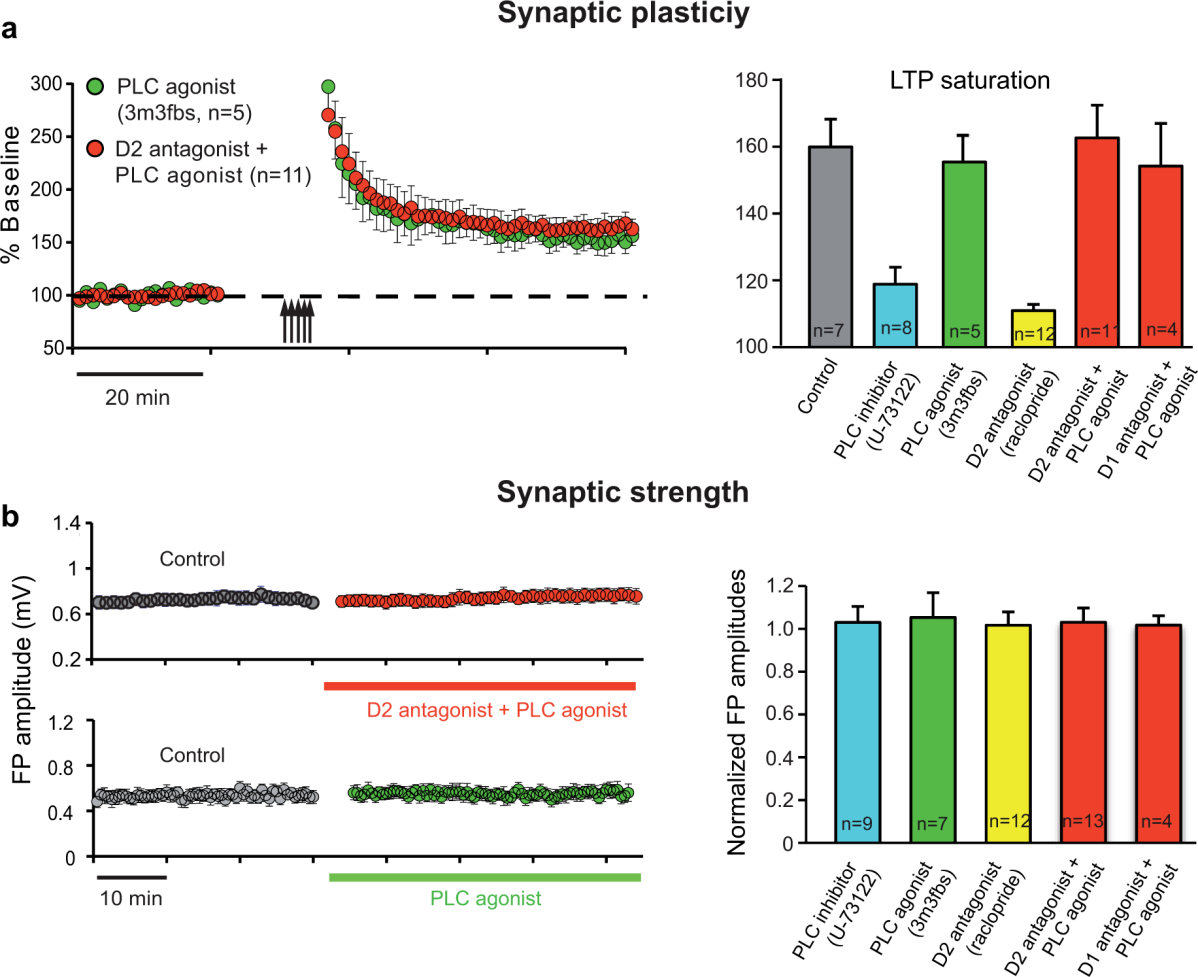
Activation of intracellular PLC pathway rescues the LTP impairment caused by DA receptor block. (a) Maximum synaptic strength of horizontal layer II/III connections, measured by saturating LTP with multiple attempts of TBS (arrows) in raclopride (D2antag) co-administered with m-3m3fbs (PLCactivator, red) resulted in normal amounts of LTP (left). m-3m3fbs alone did not affect LTP saturation (green). The histogram (right) illustrates LTP saturation for control condition (grey), PLC agonist (green), PLC antagonist (blue), D2 antagonist (yellow). D1 and D2 antagonist co-applied with the PLC agonist (red) illustrates the rescue of D1 and D2 block. (b) FP amplitudes were not affected by co-administration of D2 antagonist and PLC agonist or by PLC agonist alone (left). The histogram (right) illustrates group data of synaptic strength for all the different conditions (color code as in a). Values are calculated relative to the baseline recordings before drug application.

We further examined whether activation of the PLC signaling pathway can rescue DA antagonist-induced LTP impairment. Synaptic strength and plasticity were determined in M1 intracortical connections before and after co-application of D1 or D2 receptor antagonist together with the PLC agonist. Saturating LTP by multiple attempts of TBS in the presence of either D1 antagonist SCH23390 plus PLC agonist m-3m3fbs or D2 antagonist raclopride plus m-3m3fbs resulted in levels of LTP (154.4±12.8, N = 4 and 162.9±9.82%, N = 11 respectively; Fig 5A left) comparable to controls (160.1±8.34%, N = 7, P = 0.35 and p = 0.85 respectively; Fig 2C), and the m-3m3fbs alone (150.3±9.81%, N = 5, p = 0.42 and p = 0.68 respectively). Thus, activation of the PLC pathway rescues LTP impairment resulting from D1 or D2 receptor blockade. The histogram (Fig 5A right) emphasizes a major role of DA induced intracellular PLC activation in M1 synaptic plasticity. Baseline synaptic transmission was not affected by D1 antagonist SCH23390, D2 antagonist raclopride and PLC agonist m-3m3fbs alone [15], or m-3m3fbs co-administered with either SCH23390 or raclopride (Fig 5B left). The extracellular FPs remained unchanged in shape and amplitude. The histogram in Fig 5B (right) illustrates drug effects as relative change to control condition (before drug application) (PLC agonist: 1.03±0.113, N = 7, P = 0.85; PLC antagonist: 1.01±0.07, N = 9, P = 0.96; D2 antagonist: 0.995±0.061, N = 10, P = 0.95; D1 antagonist & PLC agonist: 0.99±0.11, N = 4, P = 0.60: D2 antagonist & PLC agonist: 1.01±0.065, N = 13, P = 0.96).

## Discussion

Dopaminergic neurons in the VTA, the major midbrain nucleus projecting to M1, play a key role in motor skill learning [16]. Our previous study showed that D1 and D2 receptor antagonists in M1 both impair motor skill learning and LTP formation [15]. To gain insight into the cellular mechanisms underlying the seemingly parallel effects of D1 and D2 receptor antagonists on LTP, we examined the intracellular pathways, specifically PKA and PLC signaling in M1 using specific inhibitors infused directly into the M1 forelimb area. The results support the hypothesis that dopaminergic signaling in M1 activates the intracellular PLC pathway to enable optimal motor skill learning and M1 synaptic plasticity. We found that PLC inhibition with intact DA transmission prevents skill learning and M1 LTP, while PLC activation in the presence of D1 or D2 antagonists mitigates learning deficits and LTP impairment.

The intracellular cascades activated by DA receptor stimulation are complex and vary between brain regions, cell types, and receptor localization [22,23,24]. While DA receptors are traditionally known to modulate cAMP signaling pathways, studies with ostensibly selective agonists or antagonists suggest that PLC can be activated by D1-like receptors [24,25,26,27]. Nevertheless DA mediated PLC stimulation remains controversial [23]. Studies in knockout mice indicate that D1-like dopaminergic activation of phosphoinositide hydrolysis is independent of D1a DA receptors [28], whereas D5 receptor knockout mice have lost the ability to produce inositol phosphate or diacylglycerol messengers after stimulation with D1-like receptor agonists [26]. Therefore, emerging evidence indicates that PLC activity can be mediated through stimulation of the D5 receptors [24,29]. Further, immunolabeling shows immunoreactivity for D1a, D2, and D5 receptors in the pyramidal tract neurons in the motor cortex [30]. As the D1-family antagonist SCH23390 does not discriminate between D1 and D5 receptors, we cannot rule out a role for D5 PLC activation in M1.

The results of this study demonstrate that blocking D2 receptors impaired skill acquisition and reduced long-term potentiation (LTP) within M1, a form of synaptic plasticity critically involved in skill learning. These results are in agreement with human studies showing that D2 receptor block abolishes neuroplasticity in the human motor cortex [35], and animal studies that suggest D2-like receptors can reinforce LTP expression by suppressing the induction of depotentiation [36]. The finding that PLC activity rescues LTP impairment resulting from D2 receptor blockade raises the possibility that D2 receptor activity acts to enhance the PLC signaling via a novel pathway. D2 receptor stimulation has been shown to mobilize Ca^2+^ stores via activation of a PLC pathway leading to calcineurin-dependent reduction of L-type Ca^2+^ currents[37]. Furthermore, George and coworkers reported that PLC signaling can occur via activation of a D1-D2 heterodimer [12,13,31,32,33]. While the selectivity of the D1-D2 heteromer-selective agonist (SKF83959) used in these studies has been challenged [23,34], studies using subtype selective ligands suggest that concurrent stimulation of D1 and D2 subtypes is coupled to calcium signaling [34].

D1 and D2 synergism has been reported to be a requirement for a variety of motor behaviors but the mechanism for this interaction remained obscure. In the PFC, which also receives DA innervation from the VTA, the induction of LTD and LTP of glutamatergic synapses is modulated by DA. DA acts through both D1 and D2 receptors to lower the threshold for the induction of this LTD [38]. Conversely, prestimulation of D1 and D2 receptors converts NMDA receptor-independent LTD to LTP [39]. D1 and D2 receptor stimulation in the nucleus accumbens is required LTD [42]. for the expression of locomotor activity [40,41], and reward-mediated processes, and concurrent D1 and D2 receptor activation had a cooperative effect [40,42]. Behavioral synergism between D1 and D2 receptors in the striatum promotes motor stereotypy [43]. This well documented behavioral synergy between D1 and D2 receptors could result from the formation of heterooligomers. Although heteromer formation in M1 is speculative and needs further elucidation it could open novel possibilities for selective pharmacological modulation of the DA-PLC pathway.
